# Exploring the causal association between epigenetic clocks and menopause age: insights from a bidirectional Mendelian randomization study

**DOI:** 10.3389/fendo.2024.1429514

**Published:** 2024-08-23

**Authors:** Ling Wang, Shuling Xu, Rumeng Chen, Yining Ding, Menghua Liu, Chunyan Hou, Zhu Wu, Xiaoju Men, Meihua Bao, Binsheng He, Sen Li

**Affiliations:** ^1^ Hunan Provincial Key Laboratory of the Research and Development of Novel Pharmaceutical Preparations, School of Pharmaceutical Science, Changsha Medical University, Changsha, China; ^2^ School of Life Sciences, Beijing University of Chinese Medicine, Beijing, China; ^3^ The Hunan Provincial Key Laboratory of the TCM Agricultural Biogenomics, Changsha Medical University, Changsha, China

**Keywords:** epigenetic clocks, DNA methylation, menopause, Mendelian randomization, causal association

## Abstract

**Background:**

Evidence suggests a connection between DNA methylation (DNAm) aging and reproductive aging. However, the causal relationship between DNAm and age at menopause remains uncertain.

**Methods:**

Employing established DNAm epigenetic clocks, such as DNAm Hannum age acceleration (Hannum), Intrinsic epigenetic age acceleration (IEAA), DNAm-estimated granulocyte proportions (Gran), DNAm GrimAge acceleration (GrimAgeAccel), DNAm PhenoAge acceleration (PhenoAgeAccel), and DNAm-estimated plasminogen activator inhibitor-1 levels (DNAmPAIadjAge), a bidirectional Mendelian randomization (MR) study was carried out to explore the potential causality between DNAm and menopausal age. The primary analytical method used was the inverse variance weighted (IVW) estimation model, supplemented by various other estimation techniques.

**Results:**

DNAm aging acceleration or deceleration, as indicated by Hannum, IEAA, Gran, GrimAgeAccel, PhenoAgeAccel, and DNAmPAIadjAge, did not exhibit a statistically significant causal effect on menopausal age according to forward MR analysis. However, there was a suggestive positive causal association between age at menopause and Gran (Beta = 0.0010; 95% confidence interval (CI): 0.0004, 0.0020) in reverse MR analysis.

**Conclusion:**

The observed increase in granulocyte DNAm levels in relation to menopausal age could potentially serve as a valuable indicator for evaluating the physiological status at the onset of menopause.

## Introduction

With the advancement of technology, biological age can be measured using various molecular or phenotypic biomarkers. For instance, research has found that the epigenetic clock, based on DNA methylation (DNAm) values and mathematical algorithms (1), can accurately quantify human aging (2), including reproductive senescence (3, 4) and immunosenescence (2). DNAm, as a significant epigenetic modification, primarily entails methylation at the fifth carbon position of cytosine, commonly observed in cytosine-guanine dinucleotides (CpG) in mammals (5, 6). Epigenetics typically includes modifications that influence the expression of genes (7, 8), and DNAm plays an important role in the development of various diseases (9–14). The commonly used epigenetic clocks include DNAm Hannum age acceleration (Hannum), Intrinsic epigenetic age acceleration (IEAA), DNAm-estimated granulocyte proportions (Gran), DNAm GrimAge acceleration (GrimAgeAccel), DNAm PhenoAge acceleration (PhenoAgeAccel), and DNAm-estimated plasminogen activator inhibitor-1 levels (DNAmPAIadjAge) measures (15, 16). Moreover, in recent years, there has been a heightened emphasis on understanding how DNAm impacts various physiological processes, with a particular focus on reproductive aging in women (17, 18). While previous studies have linked changes in DNAm patterns to conditions like cancer and neurological diseases, additional research is required to clarify the role of DNAm in the menopausal transition (19–22). We aim to investigate the causal connection between DNAm and menopausal age using established DNAm-based epigenetic clocks.

Menopause occurs due to the depletion of the limited supply of ovarian follicles, leading to a decrease in the production of the ovarian hormones progesterone and estrogen (23). Confirmation of the natural menopause typically occurs after 12 consecutive months of amenorrhea, in the absence of pathological causes (24). Nevertheless, research suggests that several factors, such as age at menarche, parity, body mass index, oral contraceptive use, alcohol consumption, smoking, and level of physical activity, often influence the onset age of menopause (25, 26). Genetic variables play a significant role in influencing menopause (27, 28), with genetic factors accounting for only 47% of the onset age, indicating that non-genetic factors also influence this hormonal aging process (29). For example, Lu et al. found that women undergoing early menopause exhibit higher levels of methylation in Alu and long interspersed nuclear element 1 (LINE-1) repetitive sequences, whereas those experiencing later menopause display lower levels of methylation (3). Conversely, data supports a nonlinear “U-shaped” relationship between the number of menstrual cycles in females and LINE-1 methylation (30). There is currently insufficient systematic study on the impact of DNAm on menopause age, and the available data does not prove a causal relationship between DNAm and menopause.

Studies employing Mendelian randomization (MR) aim to assess whether the observed association between an exposure and an outcome may suggest causation. This is achieved by investigating whether genetic variations associated with the exposure are also linked to the outcome (31–34). Our goal is to investigate the existence of a causal link between DNAm biomarkers and menopausal age, as well as to uncover potential underlying mechanisms. By using MR validation to evaluate the impact of DNAm on women’s menopausal transition, we may better comprehend the intricate relationship between reproductive aging and epigenetic regulation, providing a scientific basis for better understanding and treatment of women’s menopause.

## Methods

### Study design

In this study, bidirectional MR analyses were conducted. Initially, forward MR analysis utilized data from genome-wide association study (GWAS) datasets with six DNAm phenotypes as the exposure variables and menopause (age at onset) as the outcomes. Subsequently, reverse MR was performed to investigate the causal associations between the exposure variable (menopause (age at onset)) and the outcome variables (six DNAm phenotypes).

### Data sources

In the bidirectional MR analysis, a range of summary datasets were employed (refer to **Supplementary Table 1**). The GWAS data for the six DNAm phenotypes were extracted from the study by McCartney et al. (15). Additionally, the GWAS data for menopause (age at onset) were obtained from the study by Loh et al. (35).

### Statistical method

Instrumental variables (IVs) were chosen based on specific criteria, including: (1) IVs and exposure were significantly associated; (2) identification of independent IVs through clumping within a 10 Mb window and linkage disequilibrium (LD) of R^2^ < 0.001; and (3) the minor allele frequency (MAF) > 0.01, as previously reported (36–39). F-statistics were computed to assess the strength of IVs, with a value exceeding 10 indicating a reduced risk of weak IV bias (40).

In both forward and reverse MR analyses, we applied three statistical methods, namely the inverse variance weighted (IVW) (41), weighted median (WM) (42), and MR-Egger methods (43). The MR-Egger intercept test was employed to evaluate horizontal pleiotropy, while MR-PRESSO’s pleiotropy correction data were utilized to detect and address potential outliers. Heterogeneity was assessed through Cochrane Q statistics. Furthermore, the influence of each IV on causal relationships was examined through a ‘leave-one-out’ approach. Given that all outcomes were continuous variables, causal estimates were derived using beta coefficients and their corresponding 95% confidence intervals (CIs). These analyses were conducted using the TwoSampleMR package within the R software environment (44).

## Results

**Figure 1:**
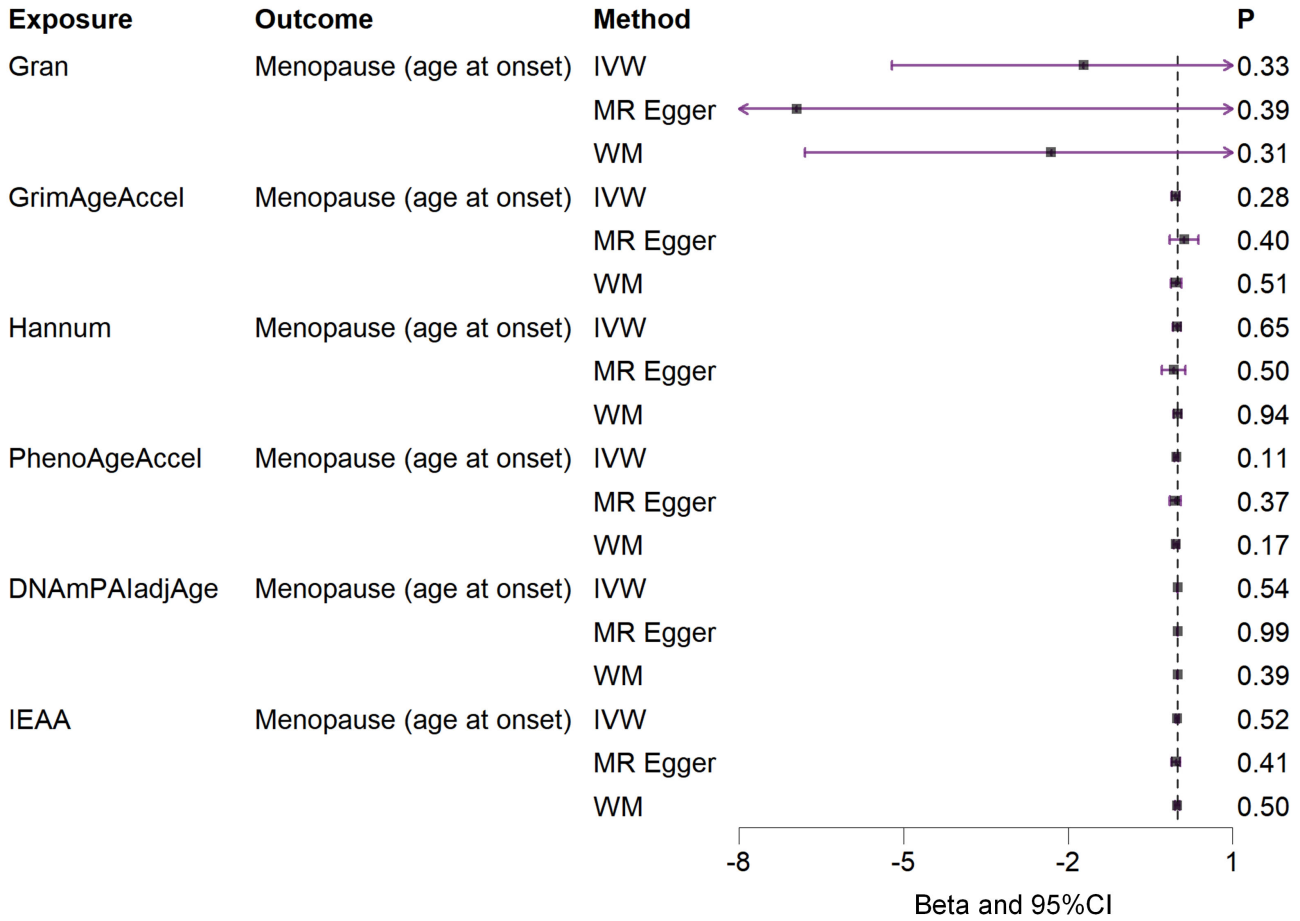
Associations between genetically predicted six DNAm phenotypes and menopause (age at onset) examined by three MR methods. DNAm, DNA methylation; Hannum, DNA methylation Hannum age acceleration; IEAA, Intrinsic epigenetic age acceleration; Gran, DNA methylation-estimated granulocyte proportions; GrimAgeAccel, DNA methylation GrimAge acceleration; PhenoAgeAccel, DNA methylation PhenoAge acceleration; DNAmPAIadjAge, DNA methylation-estimated plasminogen activator inhibitor-1 levels; MR, Mendelian randomization; IVW, inverse-variance weighted; WM, weighted median; CI, confidence interval.

**Figure 2:**
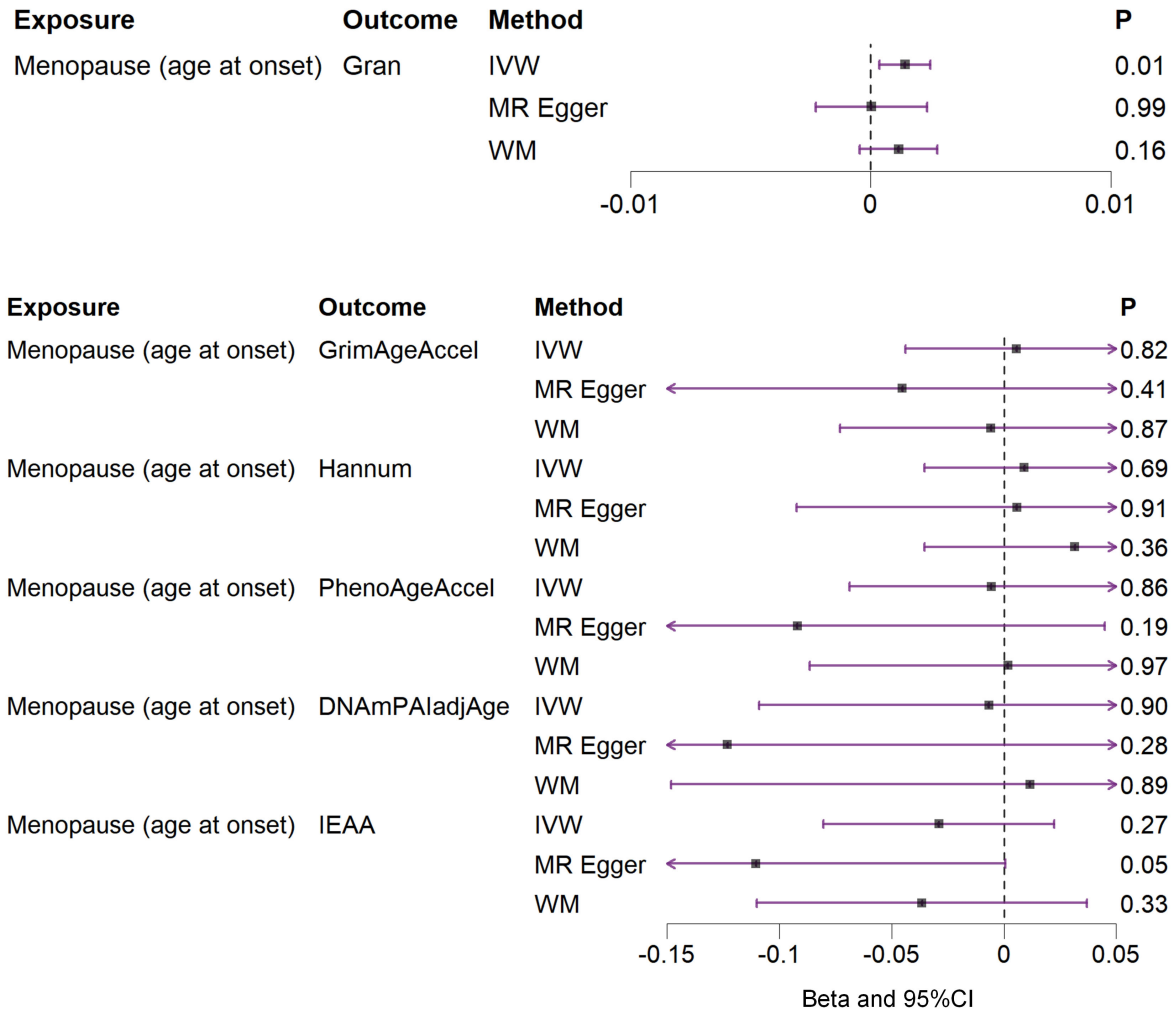
Associations between genetically predicted menopause (age at onset) and six DNAm phenotypes examined by three MR methods. DNAm, DNA methylation; Hannum, DNA methylation Hannum age acceleration; IEAA, Intrinsic epigenetic age acceleration; Gran, DNA methylation-estimated granulocyte proportions; GrimAgeAccel, DNA methylation GrimAge acceleration; PhenoAgeAccel, DNA methylation PhenoAge acceleration; DNAmPAIadjAge, DNA methylation-estimated plasminogen activator inhibitor-1 levels; MR, Mendelian randomization; IVW, inverse-variance weighted; WM, weighted median; CI, confidence interval.

**Figure 3:**
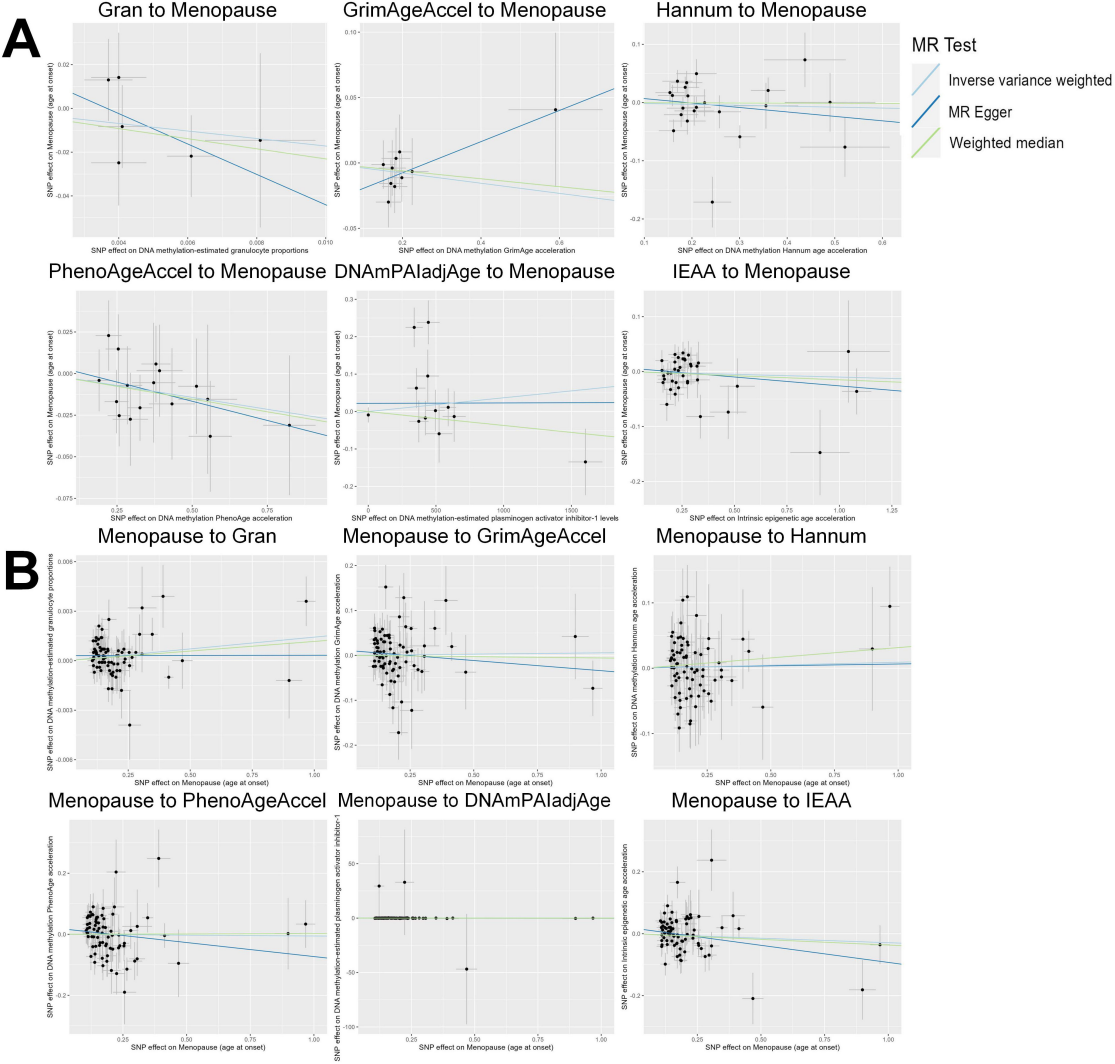
Scatter plot of the MR analyses. **(A)**: The influence of six DNAm phenotypes on the age of menopause; **(B)**: The impact of age of menopause on six DNAm phenotypes. SNP, single nucleotide polymorphism; MR, Mendelian randomization; DNAm, DNA methylation; Hannum, DNA methylation Hannum age acceleration; IEAA, Intrinsic epigenetic age acceleration; Gran, DNA methylation-estimated granulocyte proportions; GrimAgeAccel, DNA methylation GrimAge acceleration; PhenoAgeAccel, DNA methylation PhenoAge acceleration; DNAmPAIadjAge, DNA methylation-estimated plasminogen activator inhibitor-1 levels.

**Figure 4:**
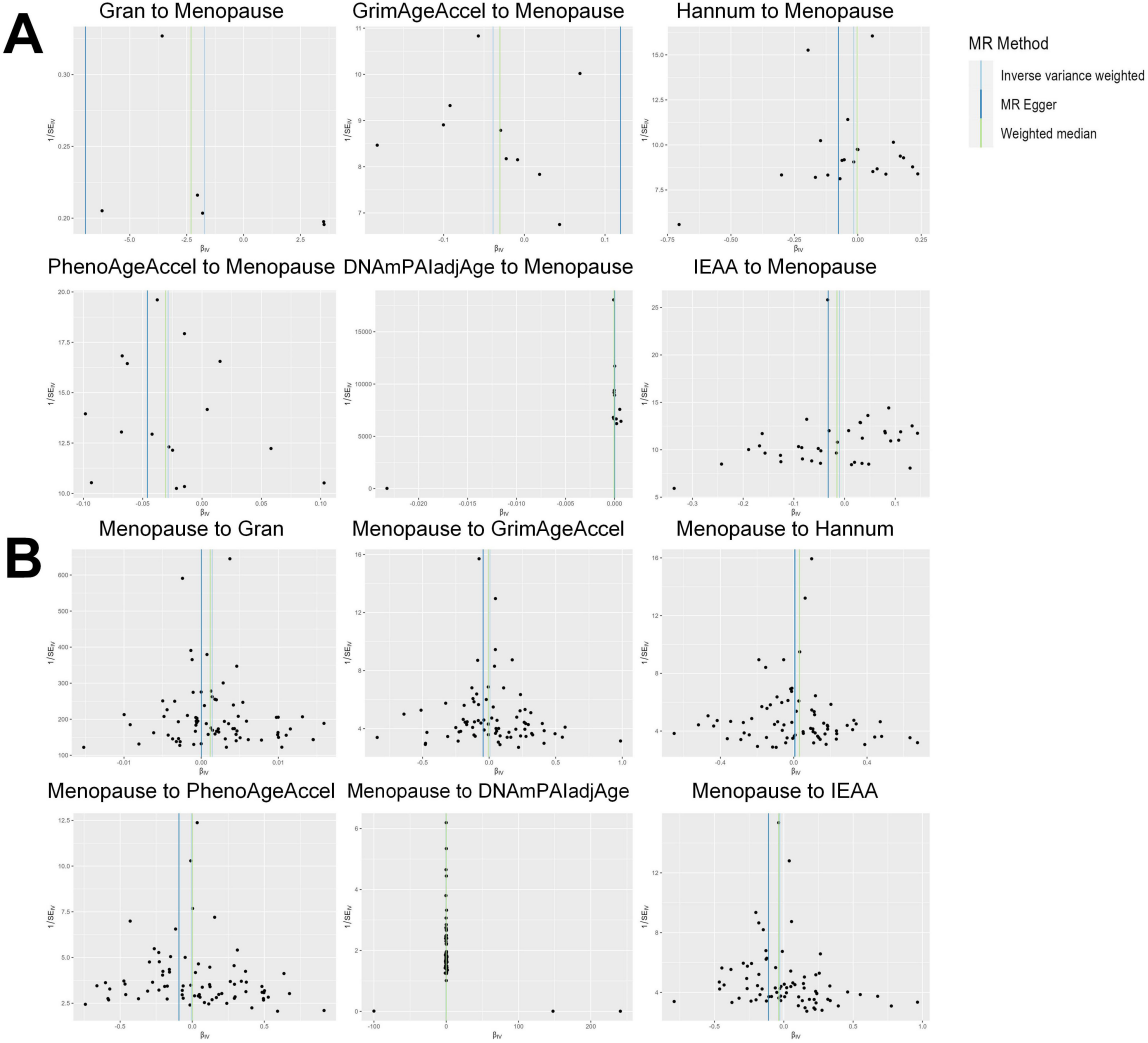
Funnel plot of the MR analyses, with each SNP acting as an IV. **(A)**: The analyses of six DNAm phenotypes on the age of menopause; **(B)**: The analyses of age of menopause on six DNAm phenotypes. SNP, single nucleotide polymorphism; IV, instrumental variable; MR, Mendelian randomization; DNAm, DNA methylation; Hannum, DNA methylation Hannum age acceleration; IEAA, Intrinsic epigenetic age acceleration; Gran, DNA methylation-estimated granulocyte proportions; GrimAgeAccel, DNA methylation GrimAge acceleration; PhenoAgeAccel, DNA methylation PhenoAge acceleration; DNAmPAIadjAge, DNA methylation-estimated plasminogen activator inhibitor-1 levels.

**Figure 5:**
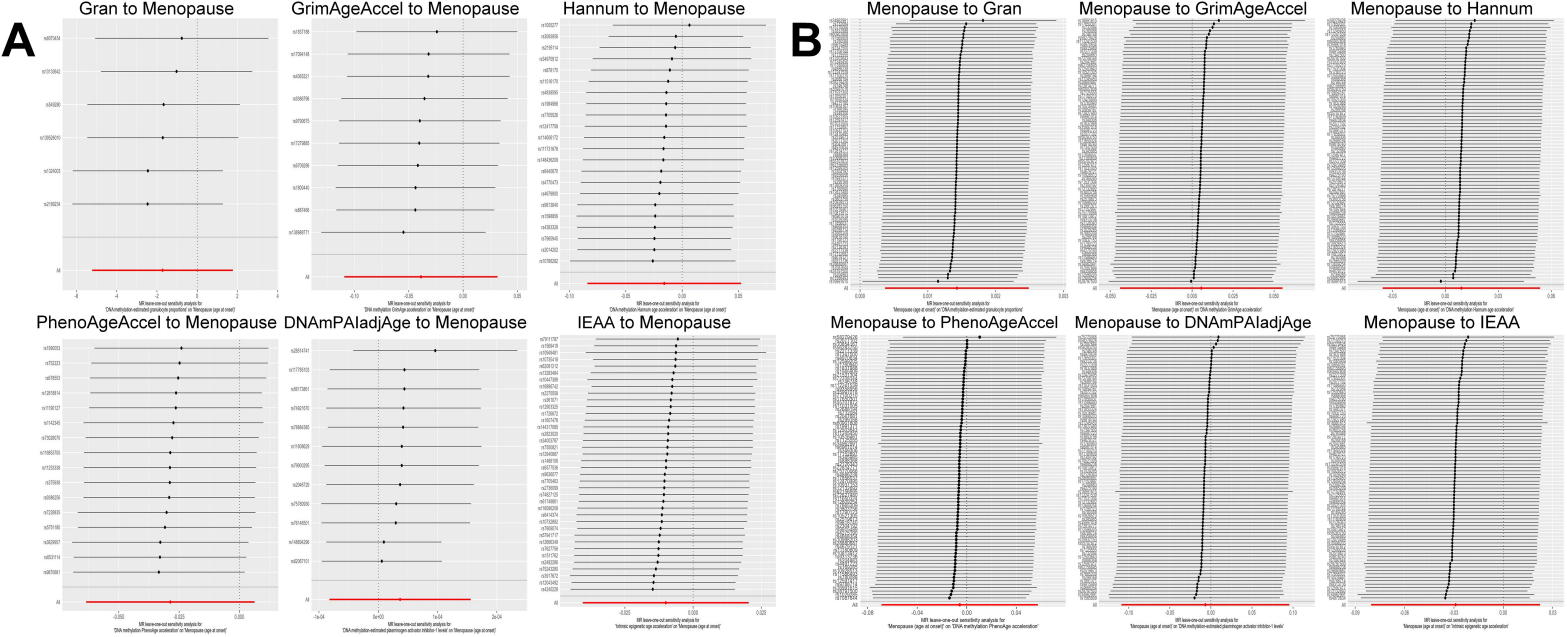
Leave-one-out sensitivity analysis by the IVW method after exclude a specific SNP from the analysis. **(A)**: The influence of six DNAm phenotypes on the age of menopause; **(B)**: The impact of age of menopause on six DNAm phenotypes. MR, Mendelian randomization; SNP, single nucleotide polymorphism; IVW, inverse-variance weighted; DNAm, DNA methylation; Hannum, DNA methylation Hannum age acceleration; IEAA, Intrinsic epigenetic age acceleration; Gran, DNA methylation-estimated granulocyte proportions; GrimAgeAccel, DNA methylation GrimAge acceleration; PhenoAgeAccel, DNA methylation PhenoAge acceleration; DNAmPAIadjAge, DNA methylation-estimated plasminogen activator inhibitor-1 levels.

Based on the rigorous inclusion and exclusion criteria applied, all IVs included in our bidirectional MR analysis, both for the forward and reverse MR approaches, demonstrated robust instrument strength. This is evidenced by F-statistic values ranging from 23.99 to 651.62 (all > 10), as outlined in **Supplementary Table 2**. The forward MR analysis showed no evidence of a causal relationship between any of the six DNAm phenotypes and menopause (age at onset) (all *P* > 0.05), as depicted in **Figure 1**; **Supplementary Table 3**. Conversely, the IVW method in reverse MR indicated a significant association between genetically predicted menopause (age at onset) and one of the six DNAm phenotypes, specifically Gran (Beta = 0.0010; 95% CI: 0.0004, 0.0020) (**Figure 2**; **Supplementary Table 4**). Consistency in the direction of the association between menopause (age of onset) and Gran was observed when employing the MR-Egger and WM methods in reverse MR (**Figure 2**; **Supplementary Table 4**). A scatter plot in **Figure 3** illustrates a positive causal relationship between menopause (age of onset) and Gran. No evidence of heterogeneity or horizontal pleiotropy between menopause (age at onset) and Gran was detected in reverse MR (**Figure 4**; **Supplementary Table 4**). Additionally, the results from MR-PRESSO indicated the absence of outliers. The leave-one-out method outcomes, as depicted in **Figure 5**, demonstrate that individual SNPs do not unduly influence the results.

## Discussion

Reproductive aging is a multifaceted process influenced by a combination of aging-related changes, environmental factors, and fluctuations in sex hormone levels (17). It is imperative to elucidate the precise causal link between menopausal age and epigenetic modifications. Conducting MR studies on DNAm and menopause provides a unique opportunity to clarify the intricate relationship between reproductive aging and epigenetic changes. The findings of this study indicated no statistically significant causal effects of epigenetic clocks on the timing of menopause. Intriguingly, our reverse causal MR analysis suggested a potential association where a later age at menopause could be linked to elevated Gran.

Women who undergo menopause at a later stage typically exhibit higher levels of sex hormones compared to those experiencing early menopause (45, 82). Fluctuations in sex hormone levels can impact DNAm. In women aged 20-30, the difference in DNAm levels between breast tissue and blood is most pronounced, and this disparity diminishes with age (46). In a comprehensive epigenome-wide study, sex hormone-binding globulin levels in teenage girls were found to be significantly associated with DNAm at three specific CpG sites and two differentially methylated regions, in contrast to men (47). Moreover, analysis of genome-wide DNAm reveals that females exhibit higher levels of DNAm than males at key loci in peripheral blood leukocytes (81.2%) (48). Therefore, we infer that sex hormone regulation could potentially account for the rise in Gran associated with delayed menopause.

We hypothesize that sex hormone levels linked to menopausal age play a role in the epigenetic alterations of immune-related granulocytes. Increased DNAm levels at CpG sites are considered a significant marker of aging (49). Studies have shown a substantial acceleration of the epigenetic age of the immune system in individuals with Parkinson’s disease, primarily associated with granulocytes (50). Therefore, we posit that the elevation in DNAm observed in granulocytes is intricately linked to the aging of the immune system. The impact of sex hormones on the immune system differs across genders, with women being more sensitive to specific infections and having a greater prevalence of autoimmune illnesses (51, 52). Therefore, the impact of female hormones on the immune system should not be overlooked. A systematic literature review indicates that progesterone levels in healthy non-pregnant women with regular menstrual cycles typically induce immunosuppression by stimulating T helper 2 cells (Th2) cytokines (53). Similarly, estrogen and its receptor signaling play a role in modulating inflammation and innate immune responses in neutrophils, with effects that vary based on gender and menopausal status (54, 55). Granulocytes, constituting approximately two-thirds of all white blood cells, represent an ideal group of immune cells for research purposes (56). Our current endeavor is to understand the mechanisms by which estrogen and progesterone impact epigenetic changes in circulating granulocytes.

DNA methyltransferases (DNMTs) may be involved in the effect of female hormones on neutrophil DNAm. DNMTs are crucial enzymes responsible for inducing DNAm, with key players being DNMT1 and DNMT3A (57). Some studies have demonstrated that the gonadal hormone 17β-estradiol (E2) can influence the methylation of CpG islands, by promoting the expression of DNMT mRNA and protein (58). For instance, microinjection of E2 into the dorsal hippocampus resulted in increased mRNA expression and protein levels of DNMT3A and DNMT3B in the hippocampus (59, 60). As individuals age, the gender disparity in DNMT expression diminishes, yet females consistently maintain methylation levels that are twice as high as males (61). Additional evidence indicates that on the initial day post-birth, females exhibit significantly higher DNMT3A mRNA expression in the amygdala, although this gender discrepancy diminishes over time (62). Nevertheless, the gender variations in DNMT activity are still a subject of debate (63, 64), and our hypothesis necessitates further validation through additional clinical investigations. Moreover, indicators of leukocyte DNAm include mitogen-activated protein kinase kinase kinase kinase 1 (MAP4K1) and homeobox A3 (HOXA3) (65). Studies have shown that progesterone promotes MAP4K1 expression in estrogen-driven breast cancer (66). The results indicate that the function of MAP4K1 is influenced by the levels of female sex hormones. Additionally, elevated levels of HOX mRNA are also typically linked to higher hormone (progesterone and estrogen) levels (67). Exploring the potential yet unexplored connection between sex hormones and HOXA3/MAP4K1 offers a novel avenue for studying the effects of menopausal age on granulocyte epigenetic changes, emphasizing the necessity for additional validation in future studies.

It is crucial to emphasize that our study did not report a significant causal link between six DNAm-based epigenetic aging markers and menopausal age. These findings might be influenced by potential confounding factors such as genetics (68), environment (69, 70), and hormones (71, 72), which also affect the age of menopause. The differentiation between menopausal age, menopause, and aging stems from the understanding that menopausal age predominantly reflects the physiological functions of the female reproductive system, whereas DNAm captures age-related alterations influenced by lifelong environmental factors and feedback mechanisms on methylation status (73, 74). While there could be a potential association between DNAm and menopausal age, assessing this influence at a population level may yield inconsistent results. Moreover, while DNAm is a crucial component of aging, it represents only a fraction of the overall process. Additional biological factors influencing menopausal age encompass gene expression, protein synthesis, and cellular signaling pathways (75–77), which may engage in intricate and not fully understood interactions with DNAm. Conversely, fluctuations in related hormones and changes in other endocrine substances due to prolonged menopause could affect the methylation of various body tissues. In future research, animal experiments could be conducted to validate whether elevated levels of hormones, DNMTs, and immune system changes are associated with increased DNAm-estimated granulocyte levels.

We are at the forefront of establishing the causal link between DNAm biomarkers and menopausal age, offering essential scientific insights to enhance our comprehension of menopause and its related conditions. Furthermore, by utilizing readily available extensive genomic and clinical epidemiological datasets, our study not only reduces experimental costs but also leverages genotype advantages to address common confounding and reverse causality issues in observational studies, thereby increasing the robustness of causal inference.

Our research design has several limitations. Firstly, our sample primarily consists of individuals of European descent, which limits the applicability of our observations in non-European women. Further validation in other ethnic groups is necessary. Secondly, the assessment of DNAm aging biomarkers may be influenced by technical issues and biological differences such as diet (78), education (79), environmental factors (80, 81), and genetic variations. These factors could introduce biases, potentially causing DNAm-based epigenetic clocks to inaccurately capture the physiological changes associated with age at menopause, despite being considered among the most reliable predictors of DNAm aging. Moreover, menopause-related diseases have not been considered as potential confounders, which may contribute to the altered metabolic and inflammatory levels in women. Lastly, our MR assessments can only determine linear causal effects, which may not fully reflect the complex relationship between DNAm and age at menopause.

## Conclusion

Our bidirectional MR analysis indicates the lack of a statistically significant causal link between DNAm aging and menopause age. Conversely, delayed menopause may be correlated with higher estimated granulocyte levels in DNAm. However, the participants in this study were all of European descent. Future research should validate these findings in diverse populations and conduct experimental studies to elucidate the underlying mechanisms. Furthermore, additional exploration of additional biomarkers is needed to better assist in the early clinical detection and intervention of female aging processes.

## Data Availability

The original contributions presented in the study are included in the article/**Supplementary Material**. Further inquiries can be directed to the corresponding authors.
