# Supplementary material for: Exploring the causal association between epigenetic clocks and menopause age: insights from a bidirectional Mendelian randomization study
Source: Front Endocrinol (Lausanne). 2024 Aug 23;15:1429514. doi: 10.3389/fendo.2024.1429514 (PMC11377254; doi:10.3389/fendo.2024.1429514)
Supplement: Supplementary file 1 [file Table1.docx]

**Supplementary Table 1.** Description of GWASs

| Phenotype | Sample size | Data download |
| --- | --- | --- |
| Menopause (age at onset) | 156364 | Dataset: GCST90029037 |
| Gran | 34470 | Dataset: GCST90014287 |
| GrimAgeAccel | 34467 | Dataset: GCST90014288 |
| Hannum | 34449 | Dataset: GCST90014289 |
| PhenoAgeAccel | 34463 | Dataset: GCST90014292 |
| DNAmPAIadjAge | 34448 | Dataset: GCST90014291 |
| IEAA | 34461 | Dataset: GCST90014290 |
| Abbreviations: GWASs: genome-wide association studies; Gran: DNA methylation-estimated granulocyte proportions; GrimAgeAccel: DNA methylation GrimAge acceleration; Hannum: DNA methylation Hannum age acceleration; PhenoAgeAccel: DNA methylation PhenoAge acceleration; DNAmPAIadjAge: DNA methylation-estimated plasminogen activator inhibitor-1 levels; IEAA: Intrinsic epigenetic age acceleration. | | |

**Supplementary Table 2.** The F-statistics of IVs

| Exposure | Number of SNPs | F-statistic | |
| --- | --- | --- | --- |
|  |  | Min | Max |
| Menopause (age at onset) | 77 | 27.54 | 651.62 |
| Gran | 6 | 25.00 | 75.94 |
| GrimAgeAccel | 10 | 24.17 | 45.49 |
| Hannum | 22 | 23.99 | 98.90 |
| PhenoAgeAccel | 16 | 24.08 | 89.39 |
| DNAmPAIadjAge | 12 | 24.45 | 162.56 |
| IEAA | 38 | 24.67 | 239.74 |
| Abbreviations: IVs: instrumental variables; SNPs: single nucleotide polymorphisms; Min: minimum; Max: maximum; Gran: DNA methylation-estimated granulocyte proportions; GrimAgeAccel: DNA methylation GrimAge acceleration; Hannum: DNA methylation Hannum age acceleration; PhenoAgeAccel: DNA methylation PhenoAge acceleration; DNAmPAIadjAge: DNA methylation-estimated plasminogen activator inhibitor-1 levels; IEAA: Intrinsic epigenetic age acceleration | | | |

**Supplementary Table 3.** Summary of forward MR of DNAm epigenetic clocks on menopausal age

| Exposure | Outcome | Method | Beta (95%CI) | *P* | Pleiotropy (*P*) | Heterogeneity (*P*) |
| --- | --- | --- | --- | --- | --- | --- |
| Gran | Menopause (age at onset) | IVW | -1.72 (-5.22～1.77) | 0.33 | 0.50 | 6.46E-01 |
|  | | MR Egger | -6.95 (-21.17～7.27) | 0.39 |  |  |
|  |  | WM | -2.31 (-6.80～2.18) | 0.31 |  |  |
| GrimAgeAccel | Menopause (age at onset) | IVW | -0.04 (-0.11～0.03) | 0.28 | 0.26 | 9.22E-01 |
|  | | MR Egger | 0.12 (-0.14～0.38) | 0.40 |  |  |
|  |  | WM | -0.03 (-0.12～0.06) | 0.51 |  |  |
| Hannum | Menopause (age at onset) | IVW | -0.02 (-0.08～0.05) | 0.65 | 0.57 | 1.12E-04 |
|  | | MR Egger | -0.08 (-0.30～0.14) | 0.50 |  |  |
|  |  | WM | -0.003 (-0.07～0.07) | 0.94 |  |  |
| PhenoAgeAccel | Menopause (age at onset) | IVW | -0.03 (-0.06～0.01) | 0.11 | 0.71 | 9.73E-01 |
|  | | MR Egger | -0.05 (-0.14～0.05) | 0.37 |  |  |
|  |  | WM | -0.03 (-0.07～0.01) | 0.17 |  |  |
| DNAmPAIadjAge | Menopause (age at onset) | IVW | 0.00004 (-0.00008～0.00016) | 0.54 | 0.55 | 3.39E-05 |
|  | | MR Egger | 0.000001 (-0.00017～0.00017) | 0.99 |  |  |
|  |  | WM | -0.00004 (-0.00012～0.00005) | 0.39 |  |  |
| IEAA | Menopause (age at onset) | IVW | -0.01 (-0.04～0.02) | 0.52 | 0.54 | 2.19E-01 |
|  | | MR Egger | -0.03 (-0.11～0.04) | 0.41 |  |  |
|  |  | WM | -0.02 (-0.06～0.03) | 0.50 |  |  |
| Abbreviations: Gran: DNA methylation-estimated granulocyte proportions; GrimAgeAccel: DNA methylation GrimAge acceleration; Hannum: DNA methylation Hannum age acceleration; PhenoAgeAccel: DNA methylation PhenoAge acceleration; DNAmPAIadjAge: DNA methylation-estimated plasminogen activator inhibitor-1 levels; IEAA: Intrinsic epigenetic age acceleration; DNAm: DNA methylation; MR: Mendelian randomization; CI: confidence interval; IVW: inverse-variance weighted; WM: Weighted median. | | | | | | |

**Supplementary Table 4.** Summary of reverse MR of menopausal age on DNAm epigenetic clocks

| Exposure | Outcome | Method | Beta (95%CI) | *P* | Pleiotropy (*P*) | Heterogeneity (*P*) |
| --- | --- | --- | --- | --- | --- | --- |
| Menopause (age at onset) | Gran | IVW | 0.001 (0.0004～0.002) | 0.01 | 0.19 | 2.13E-01 |
|  | | MR Egger | 0.00002 (-0.0023～0.002) | 0.99 |  |  |
|  |  | WM | 0.001 (-0.0004～0.003) | 0.16 |  |  |
| Menopause (age at onset) | GrimAgeAccel | IVW | 0.006 (-0.04～0.06) | 0.82 | 0.30 | 2.02E-02 |
|  | | MR Egger | -0.045 (-0.15～0.06) | 0.41 |  |  |
|  |  | WM | -0.006 (-0.07～0.06) | 0.87 |  |  |
| Menopause (age at onset) | Hannum | IVW | 0.009 (-0.04～0.05) | 0.69 | 0.94 | 2.01E-01 |
|  | | MR Egger | 0.006 (-0.09～0.10) | 0.91 |  |  |
|  |  | WM | 0.032 (-0.04～0.10) | 0.36 |  |  |
| Menopause (age at onset) | PhenoAgeAccel | IVW | -0.006 (-0.07～0.06) | 0.86 | 0.17 | 1.43E-02 |
|  | | MR Egger | -0.092 (-0.23～0.04) | 0.19 |  |  |
|  |  | WM | 0.002 (-0.09～0.09) | 0.97 |  |  |
| Menopause (age at onset) | DNAmPAIadjAge | IVW | -0.007 (-0.11～0.10) | 0.90 | 0.25 | 6.38E-01 |
|  | | MR Egger | -0.123 (-0.35～0.10) | 0.28 |  |  |
|  |  | WM | 0.012 (-0.15～0.17) | 0.89 |  |  |
| Menopause (age at onset) | IEAA | IVW | -0.029 (-0.08～0.02) | 0.27 | 0.11 | 9.78E-03 |
|  | | MR Egger | -0.110 (-0.22～0.0006) | 0.05 |  |  |
|  |  | WM | -0.037 (-0.11～0.04) | 0.33 |  |  |
| Abbreviations: Gran: DNA methylation-estimated granulocyte proportions; GrimAgeAccel: DNA methylation GrimAge acceleration; Hannum: DNA methylation Hannum age acceleration; PhenoAgeAccel: DNA methylation PhenoAge acceleration; DNAmPAIadjAge: DNA methylation-estimated plasminogen activator inhibitor-1 levels; IEAA: Intrinsic epigenetic age acceleration; DNAm: DNA methylation; MR: Mendelian randomization; CI: confidence interval; IVW: inverse-variance weighted; WM: Weighted median. | | | | | | |
